# Supplementary material for: Intraovarian, Isoform-Specific Transcriptional Roles of Progesterone Receptor in Ovulation
Source: Cells. 2022 May 5;11(9):1563. doi: 10.3390/cells11091563 (PMC9105733; doi:10.3390/cells11091563)
Supplement: Supplementary file 1 [file cells-11-01563-s001.zip › cells-1691805-Supplementary Figures.pdf]

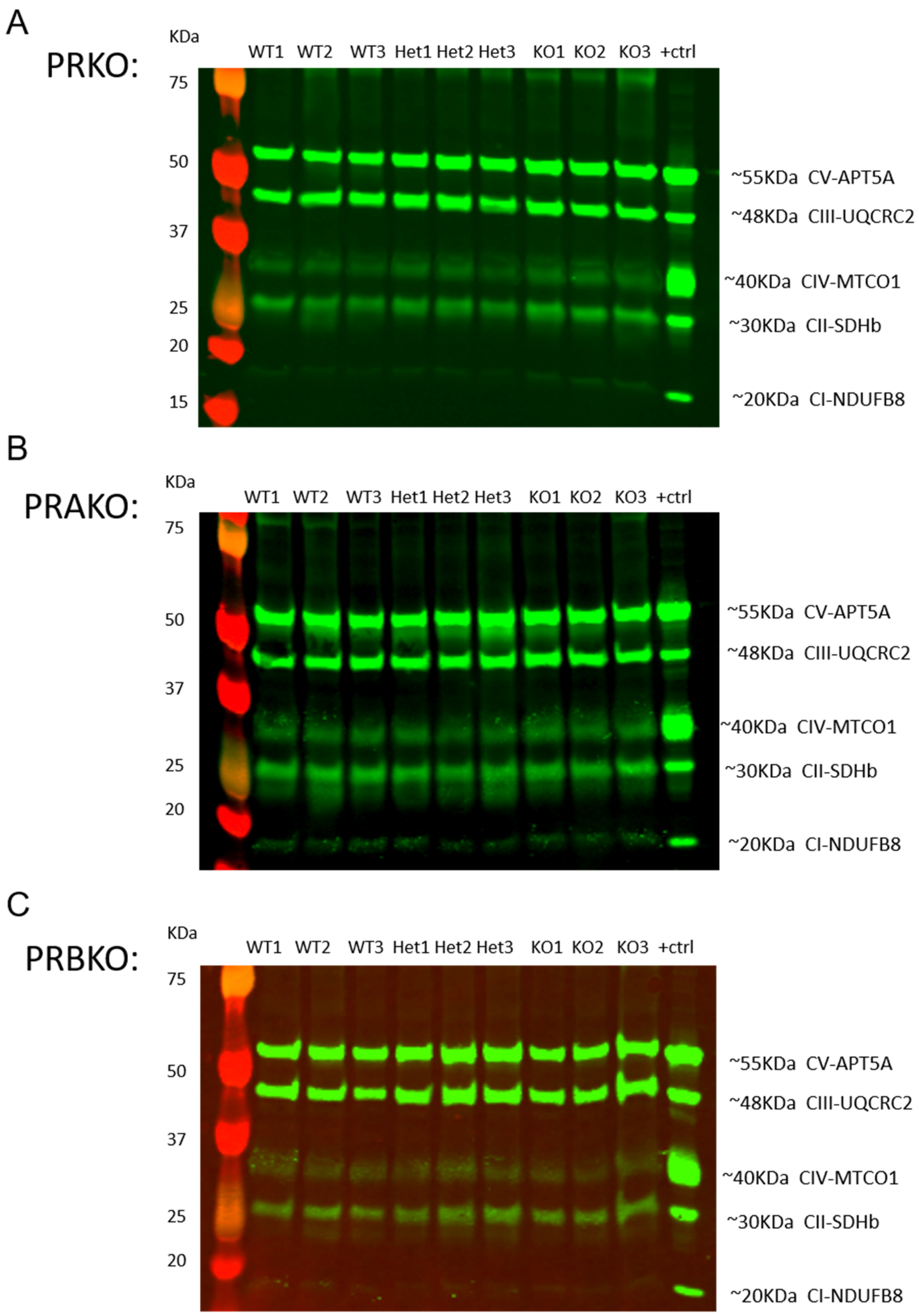

Supplementary Figure S1. Western blot of oxidative phosphorylation (OXPHOS) proteins in PRKO, PRAKO and PRBKO granulosa cells. Granulosa cells were collected at 10hrs post-hCG from (A) PRKOs, (B) PRAKOs and (C) PRBKOs as well as wildtype (WT) and heterozygous (Het) littermate controls. GC protein concentration was normalized across samples and equal amounts separated by electrophoresis. Western blot probed for APT5A, UQCRC2, MTCO1, SDHb and NDUFB8 electron transport chain complex proteins using an OXPHOS antibody cocktail. Molecular weight marker was included in far left lane and positive control (+cont) was rat heart mitochondrial protein extract.

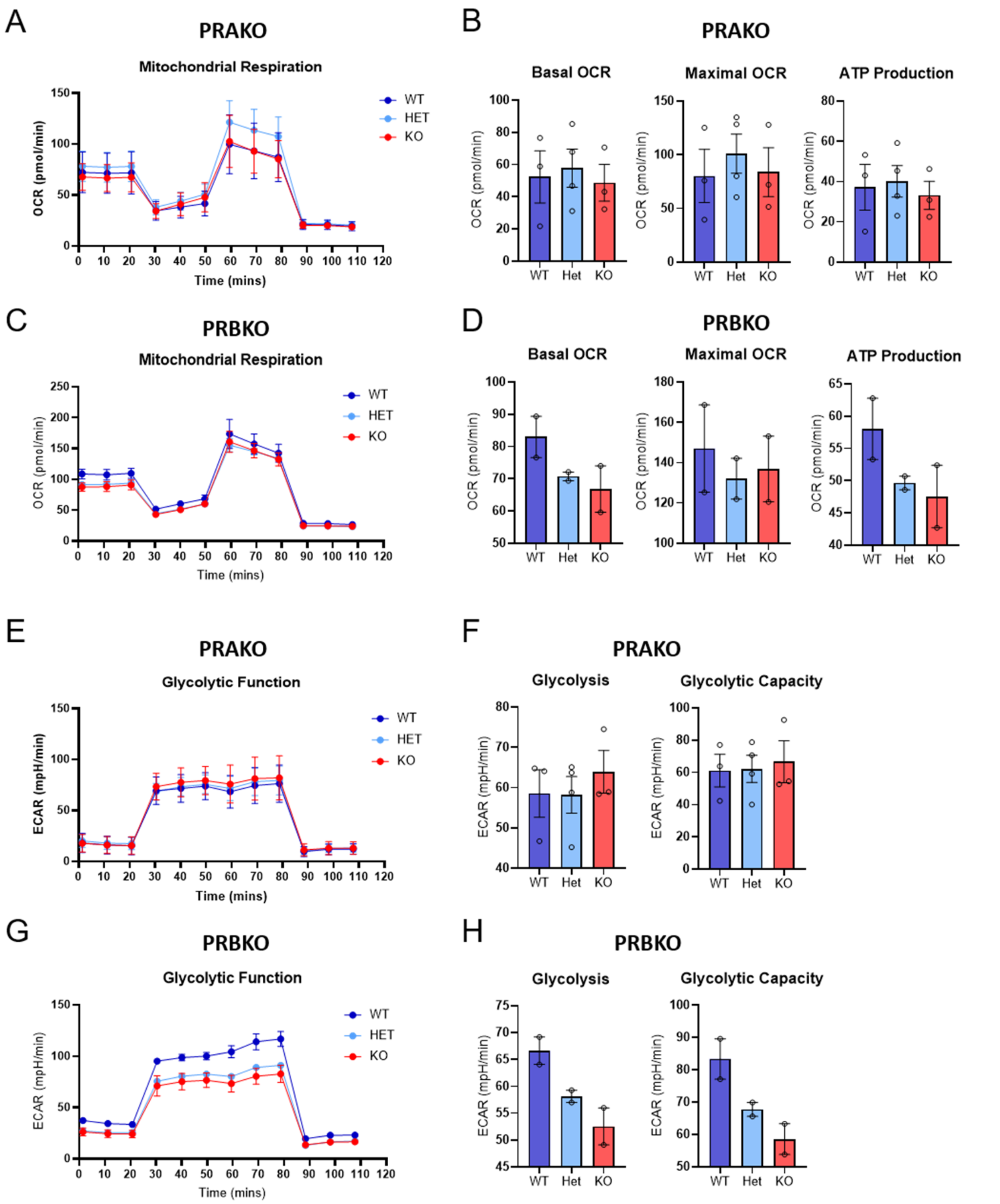

Supplementary Figure S2. Metabolism in granulosa cells from PRAKO and PRBKO mice. Granulosa cells were collected at 10hrs post-hCG from PRAKO and PRBKO mice and WT and het littermates. (A,B) mitochondrial stress test analysis of PRAKOs and (C,D) PRBKOs. (E,F) Glycolysis stress test of PRAKO and (G,H) PRBKO granulosa cells. Values represent mean ± SEM of n=2-4 biological replicates pooled from 1-3 mice. All results deemed not statistically significant by one-way ANOVA

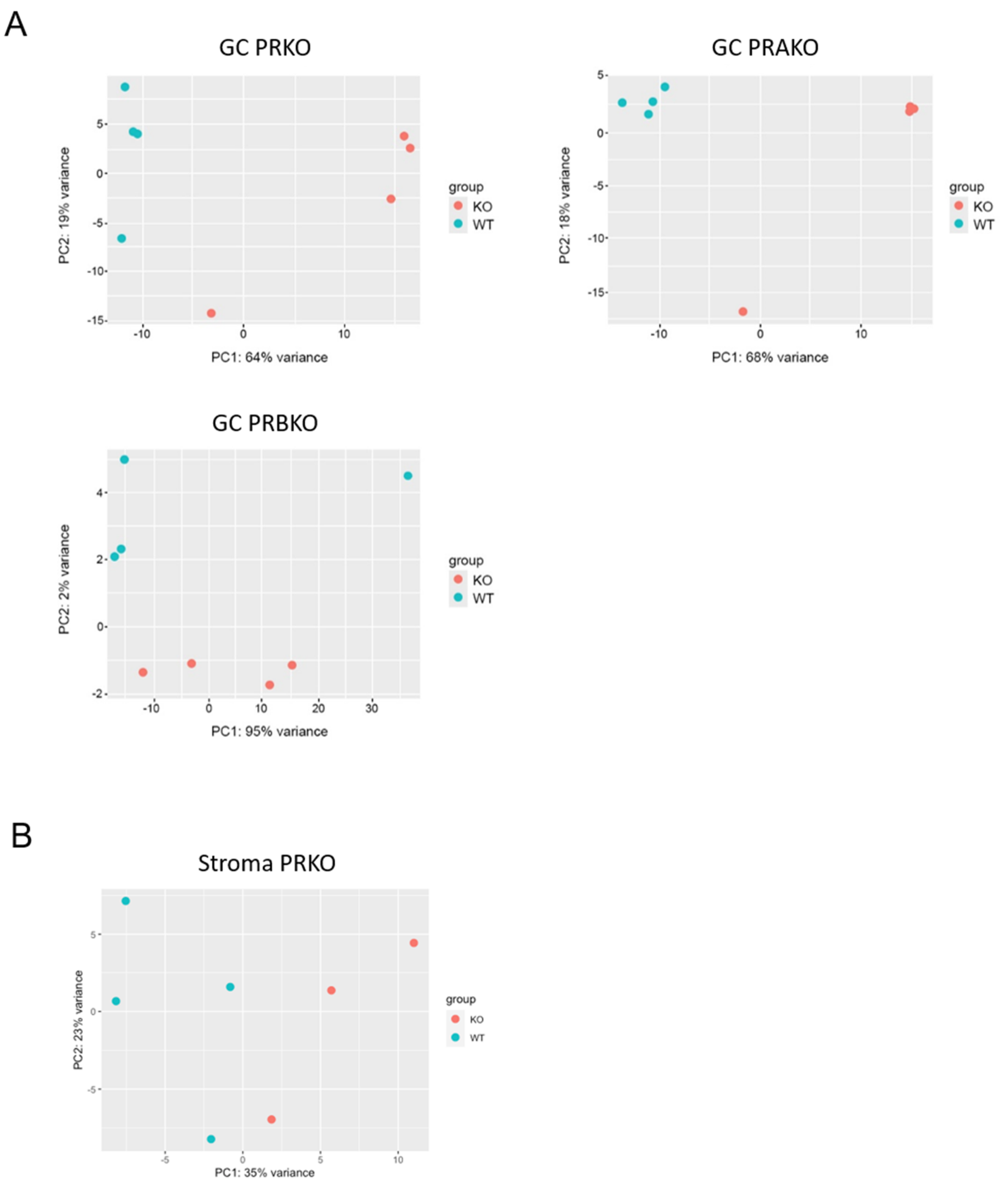

Supplementary Figure S3. Principal component analysis for RNA-seq of KO and WT samples. RNA-seq analysis was performed on (A) granulosa cells from PRKO, PRAKO and PRBKO mice and (B) stromal tissue from PRKO mice. Principle component analysis (PCA) was performed with WT (blue) and KO (red) samples plotted. Each dot represents a biological replicate (n=3-4 per genotype), each pooled from 3 mice.
